# Stick or Switch: A Selection Heuristic Predicts when People Take the Perspective of Others or Communicate Egocentrically

**DOI:** 10.1371/journal.pone.0159570

**Published:** 2016-07-20

**Authors:** Shane L. Rogers, Nicolas Fay

**Affiliations:** 1 School of Psychology and Social Science, Edith Cowan University, Perth, Western Australia, Australia; 2 School of Psychology, University of Western Australia, Perth, Western Australia, Australia; Centre for Coevolution of Biology & Culture, University of Durham, UNITED KINGDOM

## Abstract

This paper examines a cognitive mechanism that drives perspective-taking and egocentrism in interpersonal communication. Using a conceptual referential communication task, in which participants describe a range of abstract geometric shapes, Experiment 1 shows that perspective-taking and egocentric communication are frequent communication strategies. Experiment 2 tests a *selection heuristic* account of perspective-taking and egocentric communication. It uses participants’ shape description ratings to predict their communication strategy. Participants’ communication strategy was predicted by how informative they perceived the different shape descriptions to be. When participants’ personal shape description was perceived to be more informative than their addressee’s shape description, there was a strong bias to communicate egocentrically. By contrast, when their addressee’s shape description was perceived to be more informative, there was a strong bias to take their addressee’s perspective. When the shape descriptions were perceived to be equally informative, there was a moderate bias to communicate egocentrically. This simple, but powerful, selection heuristic may be critical to the cumulative cultural evolution of human communication systems, and cumulative cultural evolution more generally.

## Introduction

Interpersonal communication is a joint activity, the goal of which is to coordinate meaning across interlocutors. How this is achieved is contentious [1,2]. Classic theories emphasize the role of mentalizing [3–5]; for communication to work, speakers build and maintain a model of their addressee that is used to inform message design [3]. By contrast, for minimalist, or egocentric, accounts mentalizing plays a peripheral role; interlocutors use low-level cues (e.g., linguistic priming), available to them during interaction, to ensure effective communication [2,6,7].

The present paper examines the different strategies people use to communicate to a partner in a task where their partner’s perspective is known or unknown (Experiment 1). How frequently do speakers adopt their partner’s perspective, or retain their own egocentric perspective? Our key experiment tests a selection heuristic account of perspective-taking and egocentric communication. Derived from population-level cultural dynamics [8], it predicts that addressee-produced descriptions are compared against self-produced descriptions, and the description perceived to be more informative is adopted. This selection heuristic is tested at the individual-level (Experiment 2).

### Perspective-Taking and Egocentric Communication

By emphasizing the deliberate, strategic message adjustments speakers make to ensure the informational needs of their addressee are met, classic theories take a top-down view of interpersonal communication. To make these strategic message adjustments speakers engage in ‘audience design’ [9]: they consider the perspective of their addressee during message design, and regularly update their addressee model, to ensure their message is tailored to their addressee’s current informational needs [10,11]. Evidence that speakers build and maintain an addressee model is supported by an empirical study showing that interlocutors develop ‘conceptual pacts’, addressee-specific agreements about how to label everyday objects [12].

For minimalist accounts successful communication arises bottom-up, by participants taking advantage of the low-level cues available to them during social interaction. On this account communication is primarily egocentric, with partner adjustments occurring downstream, if at all. Minimalist accounts suggest an alternative explanation of how conceptual pacts arise and become partner-specific. Conceptual pacts can arise via low-level linguistic priming that operates during conversation [7], and can become partner-specific by particular labels becoming associated in memory with particular individuals [6]. A meta-analysis of eye-tracking studies on conceptual pacts supports this interpretation, but also identifies a small downstream partner-specific effect that is consistent with audience design [13].

Other empirical studies demonstrate partner-specific adjustments during interpersonal communication. For example, people’s beliefs about the expertise of their addressee affects message design [14,15]. Results from a spatial referential communication task suggest audience design is ubiquitous [16]. In Schober [16] participants describe the location of an object in an array (two identical circles) to a partner, who occupies the same or a different physical vantage point (explicit in the array). Participants can give an egocentric description (“it’s on my left”), or they can take their partner’s perspective (“it’s on your right”). When their partner occupies a different vantage point, participants overwhelmingly took their partner’s perspective: on 90% of trials in a non-interactive condition (imaginary partner) and slightly less often—81% of trials—in an interactive condition (co-present partner).

### Present Study

**Fig 1:**
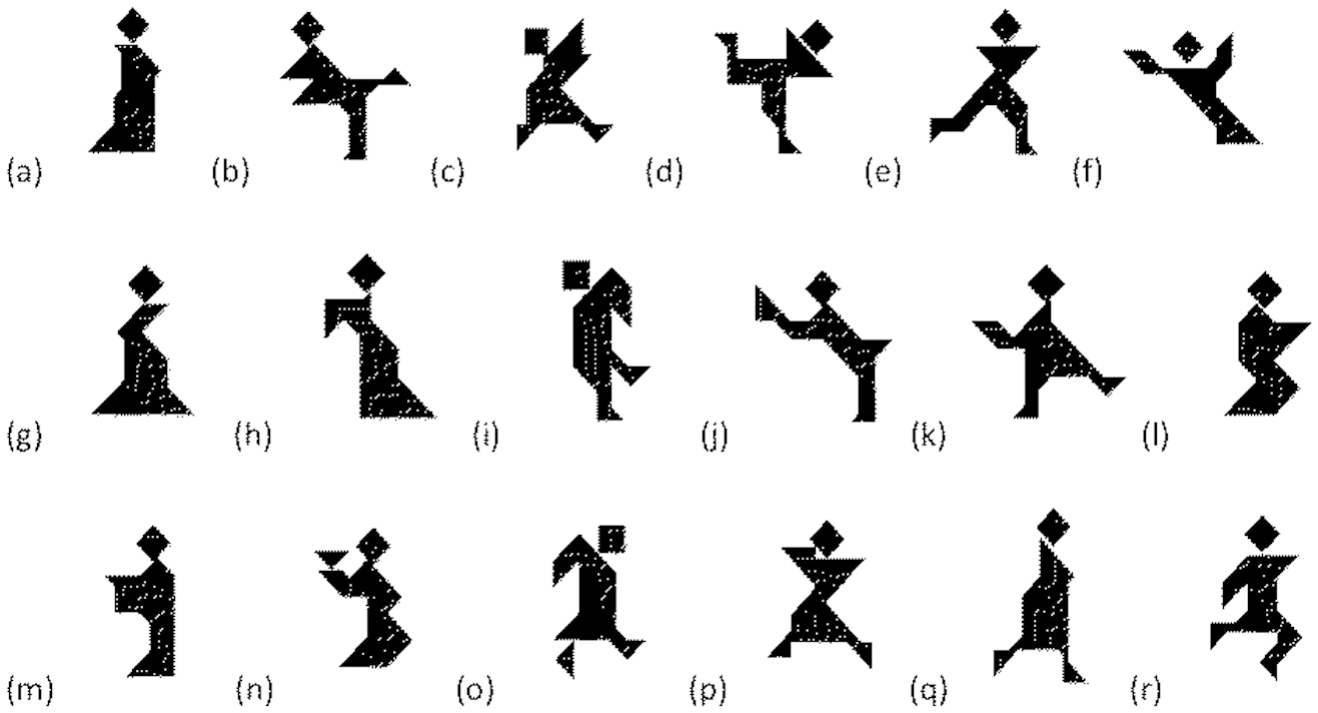
Abstract geometric shapes described by participants in Experiment 1 and 2 (shapes sampled from [17]).

If you described shape (h) from Fig 1 as “the arrow”, but your addressee described it as the “sleepwalker”, would this information change how you communicate the shape to your addressee? Would you stick with your original description, switch to your addressee’s description, or combine the descriptions? This is examined in Experiment 1. Experiment 2 forces participants to choose between the competing descriptions (personal, addressee), and uses participants’ ratings of description informativeness to predict their choice.

Experiment 1 examined the ubiquity of perspective-taking using a more complex conceptual analogue of the spatial referential communication task used by Schober [16]. In Experiment 1 we find great variation in the communication strategies employed; participants retained their egocentric perspective as often as they adopted their partner’s perspective. Experiment 2 examined a cognitive mechanism that drives perspective-taking and egocentric communication. We tested a simple *selection heuristic* account [8]. It predicts that addressee-produced descriptions are compared against self-produced descriptions, and the description perceived to be more informative is adopted.

## Experiment 1

People can and do take the perspective of others to improve communication success. Schober [16] demonstrates that adopting the perspective of one’s addressee is the dominant response, whereas Kronmüller and Barr [13] suggest a much smaller effect. In Experiment 1 participants wrote personal descriptions for a range of abstract geometric shapes (see Fig 1). Next, they wrote shape descriptions (same set of shapes) for an addressee whose perspective was either known or unknown. This two-stage process allowed us to determine the extent to which participants adjusted their communication from their personal perspective to their addressee’s perspective.

Perspective-taking in the Schober [16] study is straightforward; it involves a restricted range of conventional expressions such as, “it’s on my left” or “it’s on your right”, that are equally informative. By contrast, there are many different ways to describe the shapes used in Experiment 1 [17] (see also [11,18]). In addition, the different shape descriptions will vary in informativeness. In a more complex environment, how frequently do participants switch to their addressee’s perspective, and how frequently do they stick with their own egocentric perspective?

## Method

The Experiments reported received approval from the University of Western Australia Ethics Committee. All participants viewed an information sheet before giving written consent to take part in the study. The information sheet and consent form were both approved by the Ethics Committee.

### Participants

Eighty undergraduate psychology students (55 females, *M* = 20.70 years, *SD* = 3.90) from the University of Western Australia participated in exchange for partial course credit or payment ($10).

### Materials and Procedure

Participants produced descriptions for 18 abstract geometric shapes (see Fig 1). First, participants typed their personal description for each shape. The shapes were presented on a Microsoft word document with space below each shape for its description. The shapes were presented in a different random order for each participant. Participants were then randomly assigned to the experimental conditions: Unknown-Addressee or Known-Addressee. In the Unknown-Addressee condition participants were instructed to write a second set of shape descriptions that would allow a random other person to pick out each shape from its description. Participants were given the same set of 18 shapes (presented in a different random order), with their personal description below each shape. Participants typed their second description for each shape below their personal description.

A similar procedure was followed in the Known-Addressee condition. After typing their personal description for each shape, participants were given the shapes (in a different random order), their personal descriptions and the descriptions produced by their addressee. The descriptions were provided below each shape, with the order of personal and addressee descriptions randomized. Participants were instructed to type a second description for each shape that would allow their addressee to pick out the shape from its description. In each condition, participants completed the task at their own pace. Testing lasted approximately 30-minutes.

The same set of addressee shape descriptions were used for all participants in the Known-Addressee condition. These were selected from a corpus of personal shape descriptions collected in a previous study that used the same set of shapes [17]. Rare shape descriptions were selected to reduce the chance of the addressee shape descriptions matching the participants’ shape descriptions. A list of the addressee shape descriptions used in Experiment 1 is available in the S1 Appendix.

### Description Coding

Several categories of communication behaviour were identified. Participants in the Unknown-Addressee condition could re-use their personal shape descriptions (*Perspective Retention*), modify their original descriptions by adding information (*Perspective Modification*) or produce a novel description (*Perspective Reconceptualization*). Two additional behaviours were available to participants in the Known-Addressee condition: abandon their personal description and adopt their addressee’s description (*Perspective Shifting*), or combine their shape description with their addressee’s shape description (*Perspective Combination*). Trials where the participant’s description matched their addressee’s description were coded as *Same Perspective*.

The first author (S.L.R.) coded participants’ communication behaviour. As a check on the reliability of the coding, 108 shape descriptions (half from the Unknown-Addressee condition and half from the Known-Addressee condition) were independently coded by a second person. The Kappa statistic indicated good inter-coder agreement (Unknown-Addressee k = 0.83, k = 2, N = 54; Known-Addressee k = 0.69, k = 2, N = 54).

## Results

**Fig 2:**
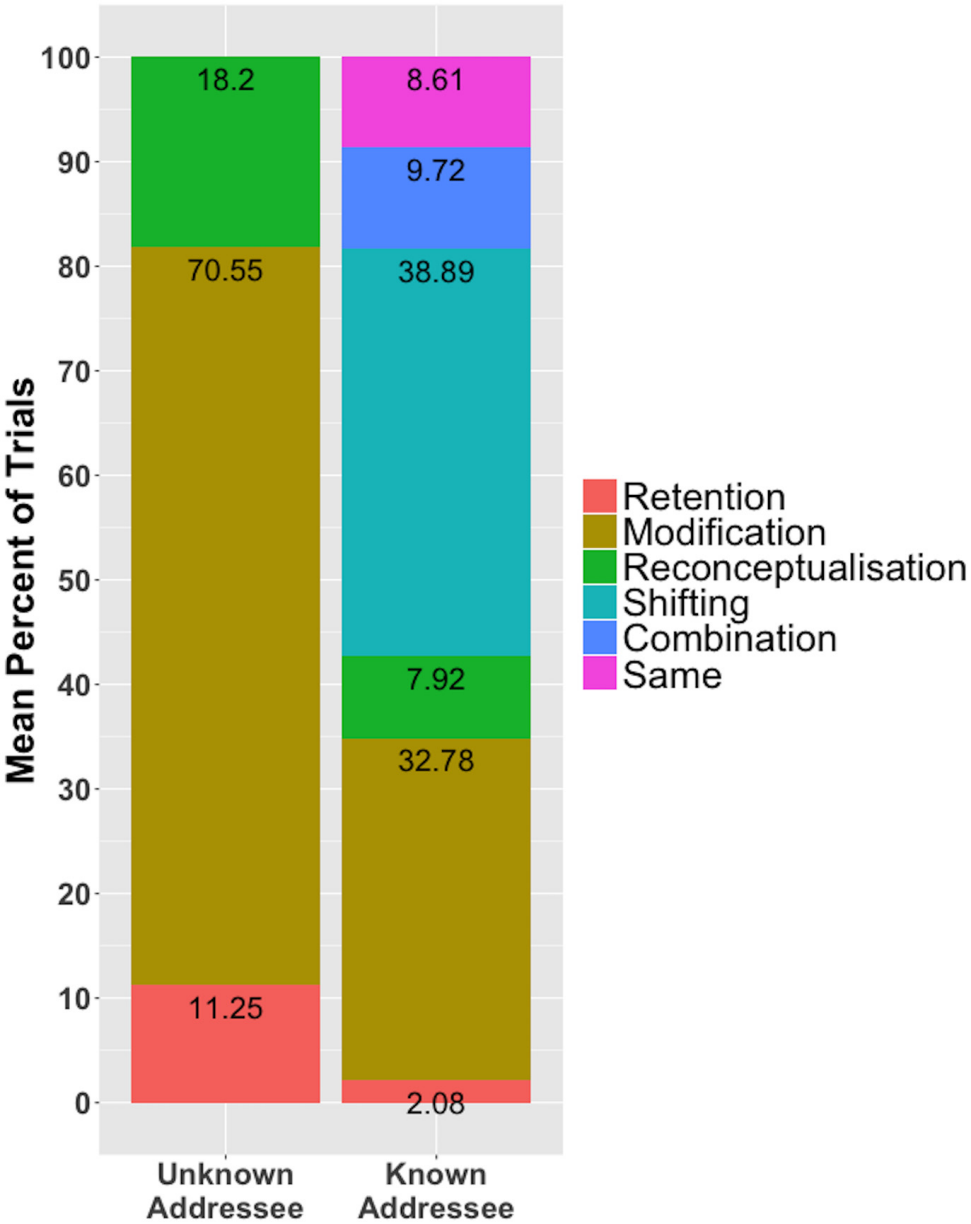
Mean percent of trials participants in the Unknown- and Known-Addressee conditions employed the different communication strategies.

Participants used a wide variety of shape descriptions; shape (h) from Fig 1 was described as “the arrow”, “zombie”, “medieval woman”, “sleepwalker”, “candle”, “nun”, “Darth Vader”, by different participants. When the addressee’s perspective was unknown, participants’ dominant response was Perspective Modification; they added information to their earlier personal description (70.55% of trials). This supports research showing that people produce more detailed descriptions for others compared to descriptions produced for themselves [17,19]. When the addressee’s perspective was known, participants’ dominant response was to switch to the perspective of their addressee (Perspective Shifting = 42.62% of trials, excluding Same Perspective trials). Note that audience-design in Experiment 1 is much less frequent compared to the spatial referential communication task [16], and egocentric communication is a frequent response (Perspective Retention plus Perspective Modification = 37.96% of trials, excluding Same Perspective trials; see Fig 2). It is important to point out that these findings were returned in an experimental setting that is conducive to audience-design; participants were under no time pressure, and there were no memory demands (personal and addressee descriptions were provided).

Using a complex conceptual referential communication task, Experiment 1 indicates that audience-design and egocentrism are common communication strategies. Our findings are limited by our use of a restricted set of rare experimenter-selected shape descriptions, and by the open-ended response format, which may have biased participants to re-use their prior shape descriptions. These design limitations are eliminated in Experiment 2.

## Experiment 2

To study the role of population-level selection dynamics on the evolution of human communication systems, Tamariz et al. [8] modelled the empirical data collected by Fay, Garrod, Roberts, and Swoboda [20]. In Fay et al. [20] participants were organized into 8-person micro-societies and played a graphical communication game, similar to the game Pictionary, with each member of their group (see also [21–23]). Initial sign variation was lost as participants interacted with the other members of their group, and aligned on a uniform inventory of sign-to-meaning mappings. Tamariz et al. [8] modelled the change in frequency of the different communication ‘variants’ in each micro-society, and found that the data was best modelled by a combination of ‘egocentric-bias’ and ‘content-bias’. When participants encountered a new sign-to-meaning mapping, they tended to reuse the sign they had used before (egocentric-bias) unless the newly encountered sign was perceived to be superior (content-bias).

Experiment 2 tests if this simple *selection heuristic* can explain interlocutors’ individual-level decisions to take their partner’s perspective, or communicate egocentrically. We use a modified version of Experiment 1 (Known-Addressee condition). This included collecting participants’ ratings of shape description informativeness and using these ratings to predict perspective-taking and egocentric behaviour. We predict that addressee shape descriptions are compared against personal descriptions, and the description perceived to be more informative is used.

## Method

### Participants

One hundred and twelve participants were recruited from the general public in exchange for $10 payment (69 females, *M* age = 40.96, *SD* = 20.14). Participants responded to a flyer posted around the University of Western Australia campus, and the surrounding area.

### Materials and Procedure

Experiment 2 followed a three-stage process. First, participants wrote personal descriptions for 18 abstract geometric shapes (same shapes used in Experiment 1). The shapes were presented on a Microsoft word document with space below each shape for its description (presented in a random order for each participant). Second, participants were presented with each shape, their personal description and their addressee’s description for the same shape (order randomized). Participants chose, by selecting a checkbox, which description (self, addressee) to return to their addressee such that their addressee could pick out the shape from its description. This procedure eliminates any benefit (cognitive or motor) associated with re-typing a previously typed shape description (Experiment 1), and forces participants to either switch to their addressee’s perspective or communicate egocentrically (i.e., stick with their personal shape description). Testing lasted approximately 30-minutes.

Another change from Experiment 1 involved using a broad range of addressee shape descriptions. Rather than restrict participants to a narrow range of experimenter-selected shape descriptions (as per Experiment 1), addressee shape descriptions were sampled from the personal descriptions produced by the previous participant in Experiment 2. That is, participant 1’s personal descriptions (participant 1 only produced personal descriptions) served as addressee shape descriptions for participant 2, whose personal descriptions served as addressee shape descriptions for participant 3, and so on.

Finally, participants were presented with each shape plus their personal shape description and their addressees’ shape description. Participants rated each shape description in terms of whether a naïve person would be able to pick out the shape from its description. Participants used a check box to indicate YES or NO to this question for their personal shape description and for their addressees’ shape description. This returned three possible outcomes for each pair of shape descriptions: Personal Description Superior (YES/NO), Addressee Description Superior (NO/YES) or Descriptions Equal (YES/YES or NO/NO).

## Results

Participants believed their shape descriptions were more informative than the shape descriptions produced by their addressee; on average they predicted that 82.94% (*SD* = 18.61%) of their personal shape descriptions, and 66.17% (*SD* = 20.09%) of their addressee’s shape descriptions, would be understood by a naïve person (paired t-test, t(111) = 6.98, p< .001, d = 0.87).

**Fig 3:**
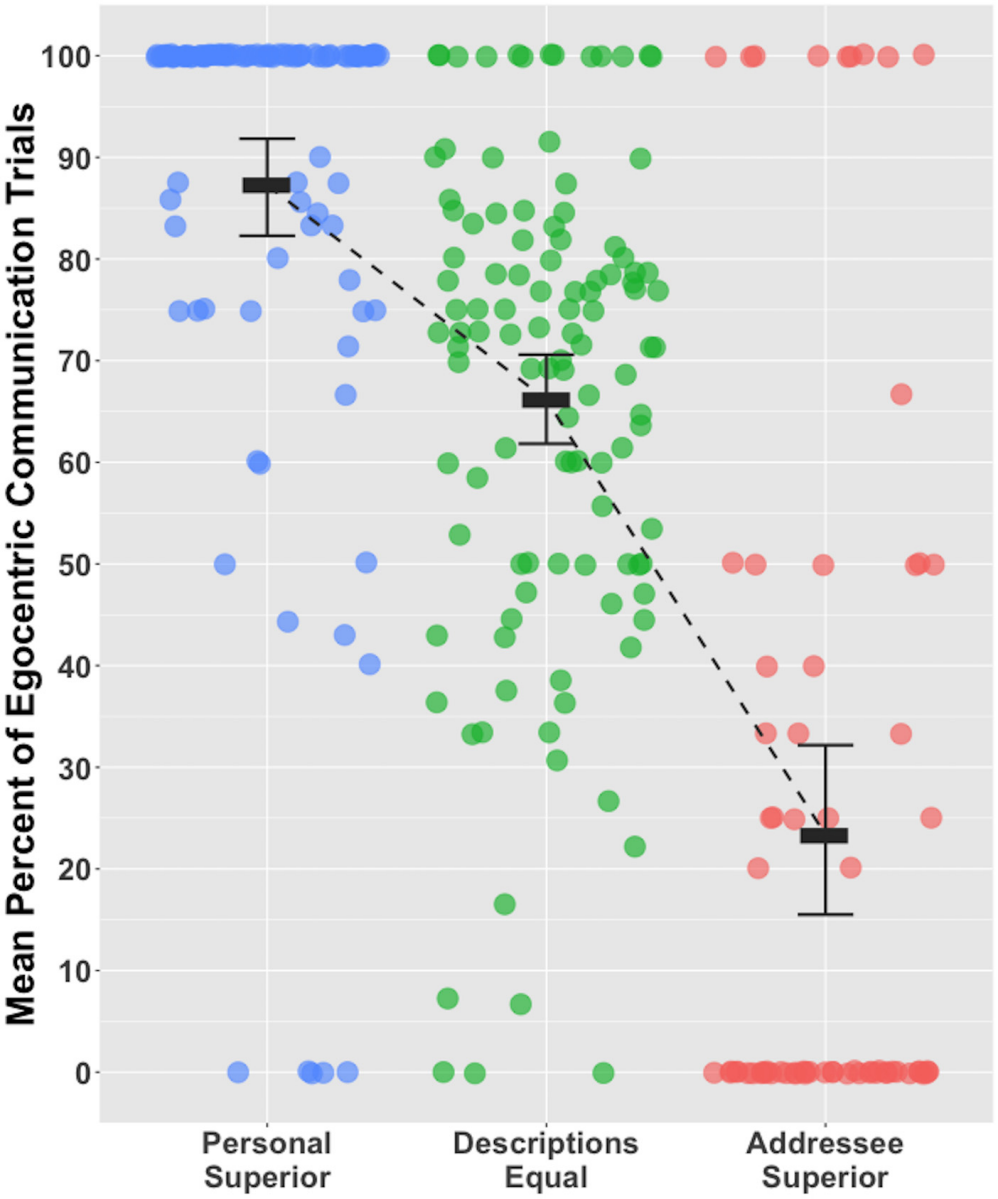
Mean percent egocentric communication by each participant when their personal shape descriptions were perceived to be more informative than their addressee’s shape descriptions (Personal Superior), when the shape descriptions were perceived to be equally informative (Descriptions Equal) and when the addressee’s shape descriptions were perceived to be more informative than their personal shape descriptions (Addressee Superior). Error bars are bootstrapped 95% confidence intervals.

Participants exhibited an egocentric-bias; on average participants chose to return their personal shape descriptions to their addressee rather than return their addressee’s shape descriptions (67.01% of trials, *SD* = 22.81; one-sample t-test, t(111) = 7.71, p< .001, d = 0.73). When rating personal and addressee shape descriptions, ‘Descriptions Equal’ was the dominant response (*M*_YES/YES_ = 56.45%, SD = 20.74%; *M*_NO/NO_ = 7.24%, *SD* = 12.14) followed by ‘Personal Description Superior’ (*M*_YES/NO_ = 26.59%, *SD* = 18.04%) and ‘Addressee Description Superior’ (*M*_NO/YES_ = 9.72%, *SD* = 12.38%). Across all ‘Personal Description Superior’ trials the dominant response was to return their personal shape description to their addressee (87.26% of trials). A weaker egocentric-bias was observed for ‘Descriptions Equal’ trials (66.14% of trials). For ‘Addressee Description Superior’ trials, the dominant response was to adopt the addressee’s perspective (egocentric communication on 23.36% of trials; see Fig 3).

Response category (Personal Superior, Descriptions Equal, Addressee Superior) was used to predict communication behaviour (perspective-taking or egocentric) in a mixed effects logistic regression model [24]. Analyses were conducted in R [25] and models were estimated using the glmer() function of lme4 [26]. P-values were obtained by likelihood ratio tests of the full model against the model without the predictors. Separate models were run for each pair of response categories (Personal Superior vs. Descriptions Equal; Personal Superior vs. Addressee Superior; Descriptions Equal vs. Addressee Superior). Following Barr, Levy, Scheepers, and Tily [27] a maximal random effects structure was specified. This included random intercepts for Participant and for Item, as well as a by-Item random slope for Response Category. As participants sometimes contributed one observation to a response category it was not appropriate to calculate a by-Participant random slope. See the S1 Dataset for the Experiment 2 data and see the S2 Appendix for the glmer model output.

Response Category affected communication behaviour. Egocentric communication was more frequent in Personal Description Superior trials compared to Description Equal trials (χ^2^(1) = 37.57, *p*< .001), and compared to Addressee Superior trials (χ^2^(1) = 58.95, *p*< .001). Egocentric communication was more frequent in Description Equal trials compared to Addressee Superior trials (χ^2^(1) = 41.47, *p*< .001). The Bayes factor associated with each of the paired tests was greater than 100 (BFs> 3.21 X 10^9^; see the S3 Appendix), indicating that the perceived informativeness of participants’ personal shape descriptions, relative to their addressee’s shape descriptions, decisively affected their communication behaviour [28].

## Discussion

When people have access to their addressee’s perspective, they often adopt their addressee’s perspective in communication. However, they do so less often on our conceptual referential task than on a simpler spatial referential communication task [16]. In fact, participants communicated from an egocentric perspective almost as often as they took their addressee’s perspective (Experiment 1). Experiment 2 examines a cognitive mechanism behind people’s decision to switch to their addressee’s perspective or stick with their own egocentric perspective.

Experiment 2 participants considered their shape descriptions to be more informative than those produced by their addressee. This is line with studies showing that people tend to believe their communication intentions are more transparent than they actually are [29–31]. When forced to choose between using their personal shape descriptions and their addressee’s shape descriptions, participants exhibited an egocentric-bias; they chose to use their own shape descriptions on 67% of trials. They did this because they believed their shape descriptions were more informative. When participants believed their personal description and their addressee’s shape description were equally informative, they showed a moderate preference to use their own shape description. When they believed their personal shape description was more informative, they showed a strong preference to use this description, and when they believed their addressee’s shape description was more informative they showed a strong preference to use that description.

This is consistent with the Tamariz et al. [8] study of variant adoption in a population of interacting agents. Here, participants preferred to use signs (i.e., drawings) they had used before (egocentric-bias) rather than adopt the sign produced by their partner, unless their partner’s sign was perceived to be superior (content-bias), in which case it was adopted. This symbiotic interplay between egocentric- and content-bias led to the selection of an inventory of sign-to-meaning mappings that was optimized for comprehension and production by a naïve second generation of learners [32,33]. This adaptive, population-level outcome can be achieved when participants’ decisions are driven by the perceived informativeness of the communication variants they encounter. Variant adoption based on informational value helps ensure the survival and propagation of variants that are optimally adapted for communication (see also copy-if-better imitation rule [34,35]). This helps explain the egocentric communication of participants in Experiment 1 and 2. Participants sacrificed the local informational needs of their addressee when their personal shape description was perceived to be more informative than their addressee’s shape description. By doing so the communication system undergoes cumulative cultural adaption at the population level [36].

## Conclusion

Perspective-taking and egocentric communication are frequent in our conceptual referential communication task (Experiment 1). On a forced choice task, egocentric communication is more frequent (Experiment 2). Participants’ decision to take their addressee’s perspective or communicate egocentrically can be explained by a simple *selection heuristic*. When their personal description is perceived to be more informative than their addressee’s description, there is a strong bias to communicate egocentrically (i.e., to stick). By contrast, when their addressee’s description is perceived to be more informative, there is a strong bias to adopt their addressee’s perspective (i.e., to switch). When the descriptions are perceived to be equally informative, there is a moderate bias to communicate egocentrically. This simple, but powerful, individual-level selection heuristic may be critical to the cumulative cultural evolution of human communication systems, and is likely to be important to cumulative cultural evolution more generally.

## Supporting Information

S1 Appendix The 18 abstract geometric shapes used in Experiment 1 and Experiment 2, plus the associated addressee shape descriptions used in Experiment 1. (DOCX)

S2 Appendix Logistic Mixed Effects Modelling Output, Experiment 2. (DOCX)

S3 Appendix Bayes Factors and Robustness Check, Experiment 2. (DOCX)

S1 Dataset Dataset, Experiment 2. (CSV)
