# Supplementary material for: Stick or Switch: A Selection Heuristic Predicts when People Take the Perspective of Others or Communicate Egocentrically
Source: PLoS One. 2016 Jul 20;11(7):e0159570. doi: 10.1371/journal.pone.0159570 (PMC4954652; doi:10.1371/journal.pone.0159570)
Supplement: S1 Appendix — (DOCX) [file pone.0159570.s001.docx]

**S1 Appendix. The 18 abstract geometric shapes used in Experiment 1 and Experiment 2, plus the associated addressee shape descriptions used in Experiment 1.**

| **Shape** | **Addressee Shape Description Used in Experiment 1** |
| --- | --- |
| 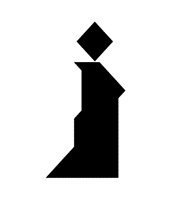 | Looks like a sorcerer who is shuffling along, he has a little collar on the coat he is wearing. |
| 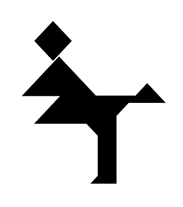 | Looks like a Piranha fish with big jaws, and a little tail. It's wearing a hat. |
| 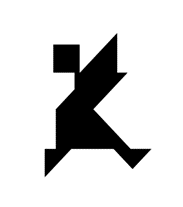 | Looks like a duck trying to take off. Its wings are flapping behind it, and it has little triangle feet. |
| 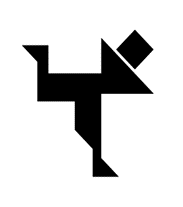 | Man doing a cartwheel with arms spread for balance. He has a leg up in the air which is bent. |
| 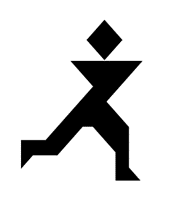 | Person stretching their calf muscle by bending down. They have short arms. |
| 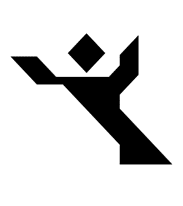 | Looks like a soccer goalkeeper diving to make a save. He has a diamond head and you can't see his feet. |
| 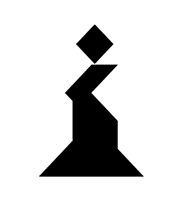 | A candle with a chunk taken out on the right side. There is wax accumulated at the bottom, with more wax accumulated on the left side. |
| 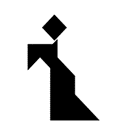 | Looks like a zombie shuffling along, with arms out in front that are bent down. |
| 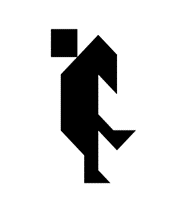 | Ninja turtle, have a shell on their back. They are doing a karate kick and their arms are out in front. |
| 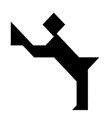 | A window washer who is leaning forward washing a window with both hands and has a spare sponge in his back pocket. |
| 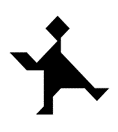 | Has a body with legs all radiating out like a starfish. The upper right arm has been chopped off so it only has four legs. |
| 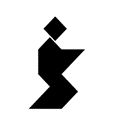 | A diver man with body poised ready to dive into water. His arms are sticking out in front and his legs are bent. |
| 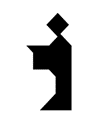 | Magician with an upturned top hat, about to pull out a rabbit. He is not wearing a cape. |
| 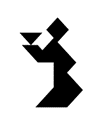 | Looks like a circus actor with her right arm bent back. On the palm of her hand she is spinning a top. |
| 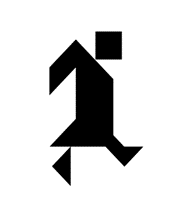 | A vampire running along with hunched shoulders, wearing a little cape. |
| 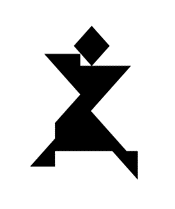 | Supermodel posing on the catwalk, she has a very thin waist and her right foot is pointed. |
| 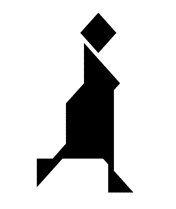 | Rollerblader, she is wearing a dress and has a big booty and little breasts. |
| 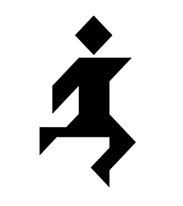 | Looks like Peter Pan with his arm stretched out in front and his legs tucked up underneath him, as if he is flying along. |
