# Supplementary material for: Stick or Switch: A Selection Heuristic Predicts when People Take the Perspective of Others or Communicate Egocentrically
Source: PLoS One. 2016 Jul 20;11(7):e0159570. doi: 10.1371/journal.pone.0159570 (PMC4954652; doi:10.1371/journal.pone.0159570)
Supplement: S2 Appendix — (DOCX) [file pone.0159570.s002.docx]

**S2 Appendix. Logistic Mixed Effects Modelling Output, Experiment 2.**

**Personal Superior vs. Descriptions Equal**

Generalized linear mixed model fit by maximum likelihood (Laplace Approximation) ['glmerMod']

Family: binomial ( logit )

Formula: Choice.n~ + Category.c + (1 | Participant) + (1 + Category.c | Item)

Data: one.2

| Predictor | Coefficient | SE | z | p |
| --- | --- | --- | --- | --- |
| (Intercept) | -2.39 | 0.19 | -12.45 | <.001 |
| Personal Superior vs. Descriptions Equal | 1.62 | 0.17 | 9.28 | <.001 |
|  |  |  |  |  |
| Model Fit: |  |  |  |  |
| AIC | 1877.2 |  |  |  |
| BIC | 1910.3 |  |  |  |
| Log-likelihood | -932.6 |  |  |  |

**Personal Superior vs. Addressee Superior**

Generalized linear mixed model fit by maximum likelihood (Laplace Approximation) ['glmerMod']

Family: binomial ( logit )

Formula: Choice.n~ + Category.c + (1 | Participant) + (1 + Category.c | Item)

Data: one.3

| Predictor | Coefficient | SE | z | p |
| --- | --- | --- | --- | --- |
| (Intercept) | -2.66 | 0.25 | -10.74 | <.001 |
| Personal Superior vs. Addressee Superior | 4.14 | 0.34 | 12.01 | <.001 |
|  |  |  |  |  |
| Model Fit: |  |  |  |  |
| AIC | 538.8 |  |  |  |
| BIC | 566.3 |  |  |  |
| Log-likelihood | -263.4 |  |  |  |

**Descriptions Equal vs. Addressee Superior**

Generalized linear mixed model fit by maximum likelihood (Laplace Approximation) ['glmerMod']

Family: binomial ( logit )

Formula: Choice.n~ + Category.c + (1 | Participant) + (1 + Category.c | Item)

Data: two.3

| Predictor | Coefficient | SE | z | p |
| --- | --- | --- | --- | --- |
| (Intercept) | -0.76 | 0.12 | -6.61 | <.001 |
| Descriptions Equal vs. Addressee Superior | 2.18 | 0.21 | 10.15 | <.001 |
|  |  |  |  |  |
| Model Fit: |  |  |  |  |
| AIC | 1778.8 |  |  |  |
| BIC | 1810.6 |  |  |  |
| Log-likelihood | -883.4 |  |  |  |
