# Supplementary material for: Stick or Switch: A Selection Heuristic Predicts when People Take the Perspective of Others or Communicate Egocentrically
Source: PLoS One. 2016 Jul 20;11(7):e0159570. doi: 10.1371/journal.pone.0159570 (PMC4954652; doi:10.1371/journal.pone.0159570)

**S3 Appendix. Bayes Factors and Robustness Check, Experiment 2.**

## Bayesian T-Test

| **Bayesian Paired Samples T-Test** | | | | | | |
| --- | --- | --- | --- | --- | --- | --- |
|  |  |  | **BF₁₀** | | **error %** | |
| Personal Superior vs. Descriptions Equal | |  | 3.843e  +9 |  | 1.924e -17 |  |
| Personal Superior vs. Addressee Superior | |  | 2.654e +12 |  | 4.421e -20 |  |
| Descriptions Equal vs. Addressee Superior | |  | 3.220e  +9 |  | 1.025e -17 |  |
|  | | | | | | |

### Inferential Plots

#### Personal Superior vs. Descriptions Equal

##### Bayes Factor Robustness Check


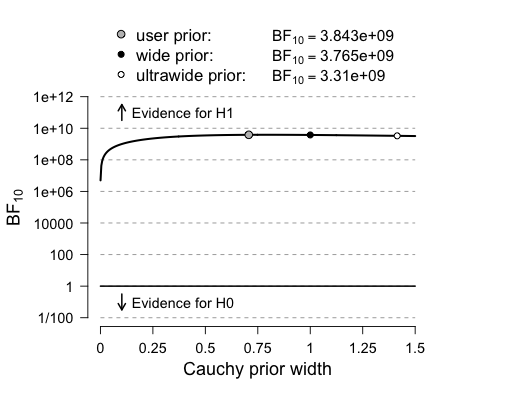


#### Personal Superior vs. Addressee Superior

##### Bayes Factor Robustness Check


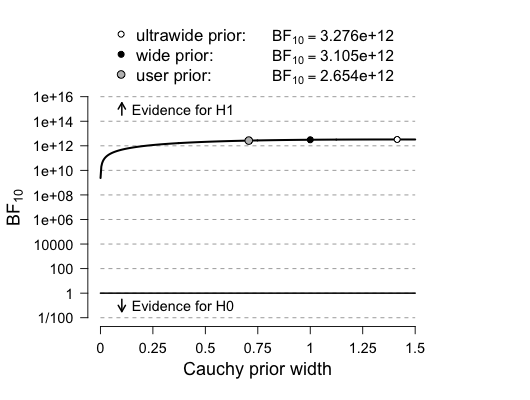


#### Descriptions Equal vs. Addressee Superior

##### Bayes Factor Robustness Check


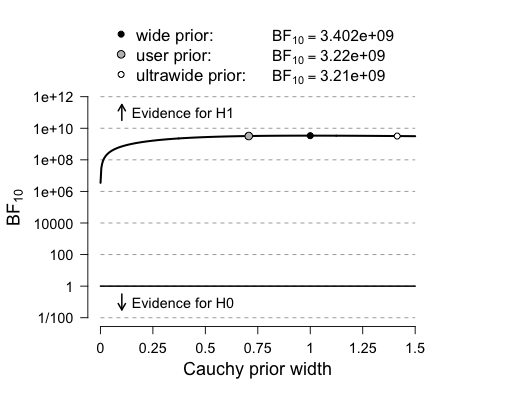

Supplement: S3 Appendix — (DOCX) [file pone.0159570.s003.docx]
